# Prevalence of overactive bladder in Chinese women: A systematic review and meta-analysis

**DOI:** 10.1371/journal.pone.0290396

**Published:** 2023-12-21

**Authors:** Shaoming Huang, Chuan Guo, Shengcheng Tai, Hongxiang Ding, Dikai Mao, Jiaguo Huang, Biao Qian

**Affiliations:** 1 Department of Urology, Ganzhou Municipal Hospital, Ganzhou, China; 2 Department of Urology, Chengfei Hospital, Chengdu, China; 3 Department of Urology, Affiliated Xiaoshan Hospital, Hangzhou Normal University, Hangzhou, China; 4 Department of Urology, The First Affiliated Hospital of Gannan Medical College, Ganzhou, China; University of Buea, CAMEROON

## Abstract

**Background:**

Overactive bladder (OAB) is a significant public health issue that adversely affects the quality of life of patients and imposes a significant socioeconomic burden, with varying prevalence rates across study populations in Chinese women. A systematic review and meta-analysis were conducted to estimate the prevalence of OAB in Chinese women.

**Methods:**

Relevant published articles on the prevalence of OAB in Chinese women were searched through July 21, 2022, using PubMed, EMbase, The Cochrane Library, China Biology Medicine (CBM), China National Knowledge Infrastructure (CNKI), WanFang Data, and VIP databases. After the independent screening of articles, data extraction, and quality assessment of included studies by two investigators, a meta-analysis was performed using Stata 16.0 software, and the prevalence was determined using a random-effects model. To identify potential sources of heterogeneity, subgroup analyses were conducted with subgroup categories including age, Body Mass Index (BMI), region, and survey year. Publication bias was assessed by visually examining the funnel plot and Egger’s test.

**Results:**

Twenty studies were included in this meta-analysis. The results of the random-effects model indicated that the prevalence of OAB in Chinese women was 14% (95% Confidence Interval: 9%–18%). The prevalence increased significantly in the past decade (from 8% in pre-2006 to 18% in 2016–2021). A prevalence (18%) was observed among women aged 31–40 compared with other age groups. The BMI range of 24–27.9 (18%) was higher than the other groups. Additionally, the prevalence of this BMI range was comparatively higher in North China and Southwest China (21%) than in Central China and East China. In addition, publication bias was observed.

**Conclusions:**

OAB incidence has increased in Chinese women over the last two decades, affecting more than 20% of women aged 31–40 years and above. With the increasing prevalence of OAB, greater emphasis has been placed on implementing preventative and control measures.

## Introduction

Overactive bladder (OAB) refers to a group of bladder diseases characterized by urinary urgency, nocturia, and urinary frequency with or without urge incontinence. OAB seriously impacts individuals’ lives, and many patients are compelled to reduce their water intake to manage OAB [1]. However, OAB affects the patient’s psychology, employment, familial relationships, body, and sexual life to varying degrees and should be given due attention by doctors and patients. According to studies, the prevalence of OAB in women aged 18 years and above varies widely, ranging from approximately 17% to 43% in Europe and the United States [2–6] to approximately 1.9% to 53.8% in Asia [7–11]. Relevant reports have also demonstrated that OAB affects patients’ quality of life (QOL) and incurs high medical costs [12]. There may be many OAB patients in China’s enormous population, and reliable estimates of OAB prevalence are necessary. This estimate may have implications for management and prevention. Owing to China’s enormous population, even modest advancements in OAB prevention management can significantly improve the health of the population. The current situation of domestic research on OAB is not systematically reviewed in the Chinese mainland; recent research on OAB focuses primarily on the effects of various treatment interventions on OAB. Common interventions are mainly drug therapy, including anticholinergics, β3-adrenoreceptor agonists, and off-label use of antidepressants in some cases [13–15]. Treating menopausal genitourinary syndrome with estrogen has also depicted promise for OAB [16]. It has been demonstrated that botulinum toxin bladder injection, tibial nerve stimulation, and acupuncture are effective treatment options [17–19]. Unfortunately, there is a lack of data on the prevalence of OAB among women in mainland China. Therefore, we performed a systematic review and meta-analysis to reliably estimate the prevalence and epidemiological characteristics of OAB among Chinese women.

## Methods

### Protocol and registration

This systematic review and meta-analysis protocol was registered in INPLASY (registration number: INPLASY202320047) and conducted according to the 2020 Preferred Reporting Items for Systematic Reviews and Meta-Analyses (PRISMA) guidelines.

### Search strategy

Relevant published articles on the prevalence of OAB in Chinese women were searched in PubMed, EMbase, the Cochrane Library, China Biology Medicine (CBM), China National Knowledge Infrastructure (CNKI), WanFang Data, and VIP databases. The retrieval method primarily relies on a combination of subject and free words. The retrieval time limit was from the inception of the database through July 21, 2022, and publication time was unrestricted. The search terms included China, Chinese, female, woman, women, overactive bladder, detrusor hyperactivity, detrusor hyperfunction, prevalence, and epidemiology. All field and medical subject heading (MeSH) terms were utilized in the PubMed database search, with Boolean operators (OR, AND, and NOT) used to join key terms. An example of the search strategy for PubMed is presented in Table 1. Other databases were referenced to the retrieval strategy of PubMed. No language restrictions were imposed, and translations were sought where necessary.

**Table 1:** The search strategy for PubMed.

Search number	Query
#1	"China"[MeSH Terms]
#2	"Chinese"[Title/Abstract]
#3	"China"[MeSH Terms] OR "Chinese"[Title/Abstract]
#4	"Female"[MeSH Terms]
#5	"Females"[Title/Abstract] OR "Women"[Title/Abstract] OR "Girls"[Title/Abstract] OR "Girl"[Title/Abstract] OR "Woman"[Title/Abstract]
#6	"Female"[MeSH Terms] OR "Females"[Title/Abstract] OR "Women"[Title/Abstract] OR "Girls"[Title/Abstract] OR "Girl"[Title/Abstract] OR "Woman"[Title/Abstract]
#7	"urinary bladder, overactive"[MeSH Terms]
#8	"overactive bladder"[Title/Abstract] OR "overactive urinary bladder"[Title/Abstract] OR "bladder overactive"[Title/Abstract] OR "overactive detrusor"[Title/Abstract] OR "detrusor overactive"[Title/Abstract]
#9	"urinary bladder, overactive"[MeSH Terms] OR "overactive bladder"[Title/Abstract] OR "overactive urinary bladder"[Title/Abstract] OR "bladder overactive"[Title/Abstract] OR "overactive detrusor"[Title/Abstract] OR "detrusor overactive"[Title/Abstract]
#10	"Prevalence"[MeSH Terms]
#11	"Prevalences"[Title/Abstract] OR "Epidemiology"[Title/Abstract] OR "Epidemiologies"[Title/Abstract]
#12	"Prevalence"[MeSH Terms] OR "Prevalences"[Title/Abstract] OR "Epidemiology"[Title/Abstract] OR "Epidemiologies"[Title/Abstract]
#13	#3 AND #6 AND #9 AND #12

### Study selection and data extraction

All published studies investigating the prevalence of OAB in Chinese women, irrespective of age or race, were included. The outcome measure was OAB prevalence. Multiple publications, meta-analyses, systematic reviews, conference papers, animal studies, and studies that lacked original data were excluded.

The articles were independently screened for inclusion and exclusion criteria by two investigators (HD and DM). Subsequently, the data of the included articles were extracted for cross-checking. In the event of disagreement, a third party (ST) was consulted for adjudication. Information about the article, such as title, author, year of publication, study type, time of the study, area of study, study subjects and sources, age, Body Mass Index (BMI), diagnostic criteria, sample size, and prevalence.

### Quality assessment

The quality evaluation criteria for cross-sectional studies, comprising 11 items as recommended by the Agency for Healthcare Research and Quality (AHRQ), were utilized to assess the quality of the articles. A score of 0 points will be assigned to responses categorized as "No" or "Unclear", while a score of 1 point will be assigned to responses categorized as "Yes" [20]. The quality of the literature was positively correlated with the score, such that a higher score indicated higher quality. The quality assessment mentioned above was evaluated through a discussion between two researchers. The evaluation was performed by two investigators (HD and DM), with a third party (ST) acting as an adjudicator in case of any disagreement. Scores ≤ 5 were considered low quality [20].

### Data analysis

Meta-analysis was conducted using Stata 16.0 software. Heterogeneity among the results of the included studies was analyzed using the Q test (test level: α = 0.1). The magnitude of heterogeneity was quantitatively evaluated using *I*^2^. According to *I*^2^ statistics, heterogeneity was categorized as low (less than 25%), moderate (25%–75%), and high (more than 75%) [21]. The prevalence of OAB in Chinese women was calculated using a random-effects model. Potential sources of heterogeneity were identified through subgroup analyses, with the subgroup categories comprising age, BMI, region, and survey year. Age was categorized as ≤ 30 and > 60 years, whereas the remaining age groups were classified by decade. Nutritional status was evaluated and grouped based on BMI, as follows: underweight (BMI < 18.5), normal weight (BMI = 18.5–23.9), overweight (BMI = 24–27.9), and obesity (BMI ≥ 28). The provinces of China were divided into seven regions: Northeast China, East China, North China, Central China, South China, Southwest China, and Northwest China. The survey years were grouped as: < 2006, 2006–2010, 2011–2015, and 2016–2021. If grouped data on subgroup categories were not available, they were excluded. Publication bias was assessed by visually examining the funnel plot and using Egger’s test. *P* < 0.05 was considered statistically significant.

## Results

**Fig 1:**
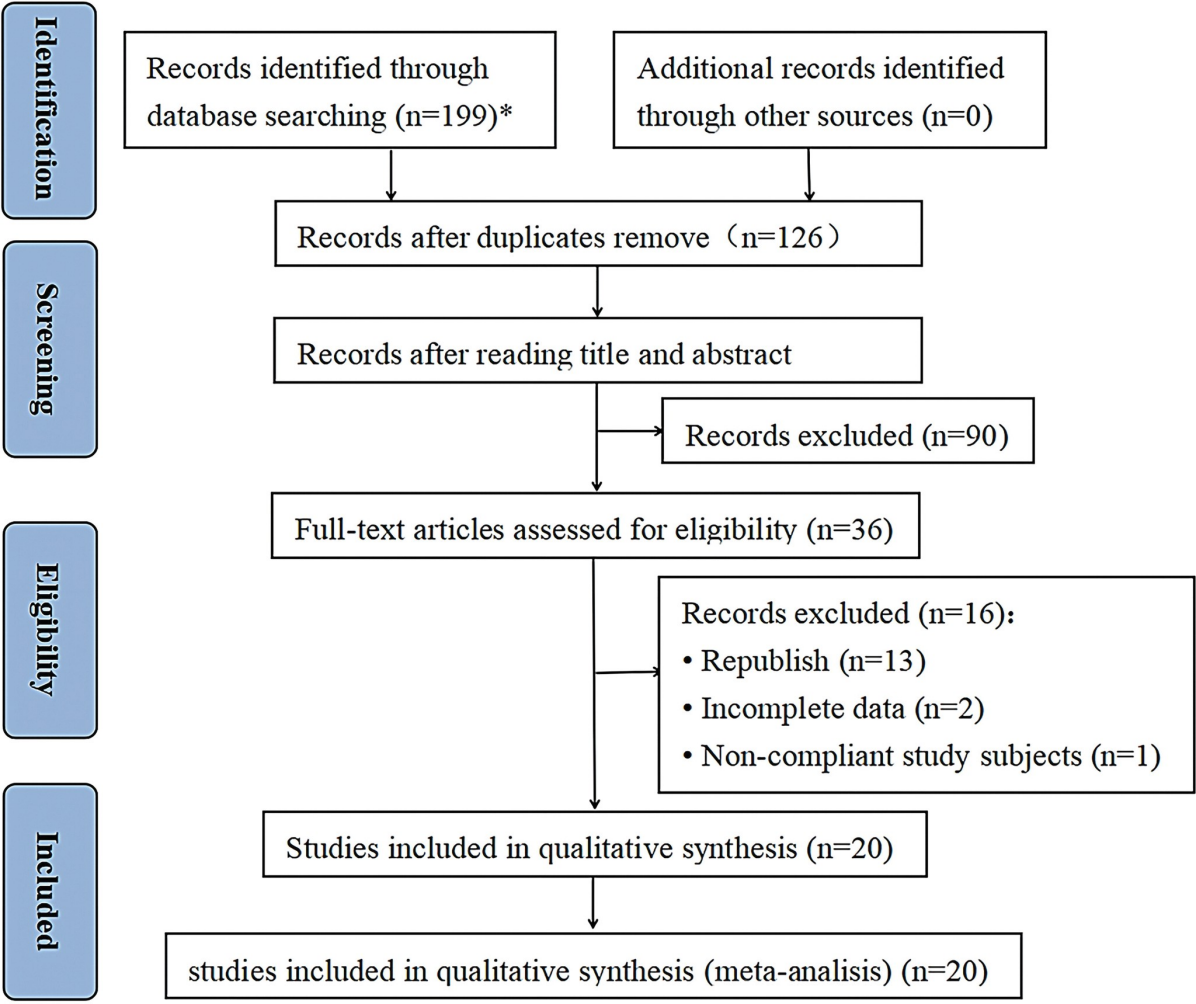
Flow diagram of the search and screening processes based on PRISMA 2020.

A flowchart of the study selection process is presented in Fig 1. In total 199 articles were retrieved from our preliminary search. After title and abstract screening, 163 articles were eliminated. Subsequently, 16 articles were excluded for various reasons, resulting in 20 eligible articles included in the analysis based on the predetermined inclusion and exclusion criteria [7–9, 22–38].

The characteristics of the 20 included studies are presented in Table 2. These studies were published between 2006 and 2021. Seven of the 20 studies were conducted in North China, five in East China, three in Central China, and one in Southwest China. The included studies had cross-sectional designs. The quality scores of all studies were higher than 5, and the overall quality of the included studies was acceptable.

**Table 2:** Characteristics of the included articles.

Reference	Region	Survey Years	Age Range	Diagnosis Standard	Cases	Sample Size	Prevalence (%)
An, F 2016	North China	2012.10–2012.11	18–97	(1)	191	2161	8.8
Chen, Y 2015	North China	2015.06.02–2015.07.20	42–59	(1)	26	351	7.4
Chuang, Y 2019	China	2015.06.02–2015.07.20	-	(1)	543	2056	26.4
Wang, Y 2011	China	2009.06–2010.02	-	(1)	449	7485	6
Wen, J 2014	Central China	2010.06–2011.02	-	(1)	125	6676	1.9
Wu, J 2016	Central China	2013.12.15–2014.06.10	-	(1)	83	590	14.1
Xing, D 2020	China	2018.07–2018.10	-	(1)	437	4761	9.18
Xu, D 2019	East China	2016.07–2016.09	21–53	(1)	102	320	32
Zhang, C 2013	North China	2010.09–2011.03	19–58	(2)	295	1070	27.57
Zhang, W 2006	East China	2002	-	(3)	377	4684	8
Zhu, L 2015	North China	2012.09–2013.12	36–76	(1)	32	304	9.43
Chen, L 2015	East China	2012.05–2012.11	20–55	(1)	122	636	19.2
Hu, Y 2021	East China	2020.07–2020.12	-	(1)	23	74	31.08
Hu, Z 2018	North China	2017.06–2018.06	19–55	(1)	276	1129	24.4
Liang, Y 2020	China	2018.09–2020.01	-	(1)	770	12701	6.1
Liao, Y 2015	Central China	2011.08–2014.11	-	(1)	115	5882	1.96
Liu, Y 2013	East China	2012.05–2012.11	22–54	(4)	81	401	20.2
Wang, Y 2010	North China	2009	18–90	(1)	112	2379	4.7
Wu, H 2020	Southwest China	2019	-	(1)	15	70	21.43
Wu, N 2017	North China	2016.04.01–2016.04.30	-	(1)	42	1000	4.2

(1) OAB symptom score questionnaire; (2) Beijing Nurses Bladder Questionnaire; (3) Bristol Female Lower Urinary Tract Symptoms Questionnaire; (4) International Consultation on Incontinence Modular Questionnaire-Female Lower Urinary Tract Symptoms

**Fig 2:**
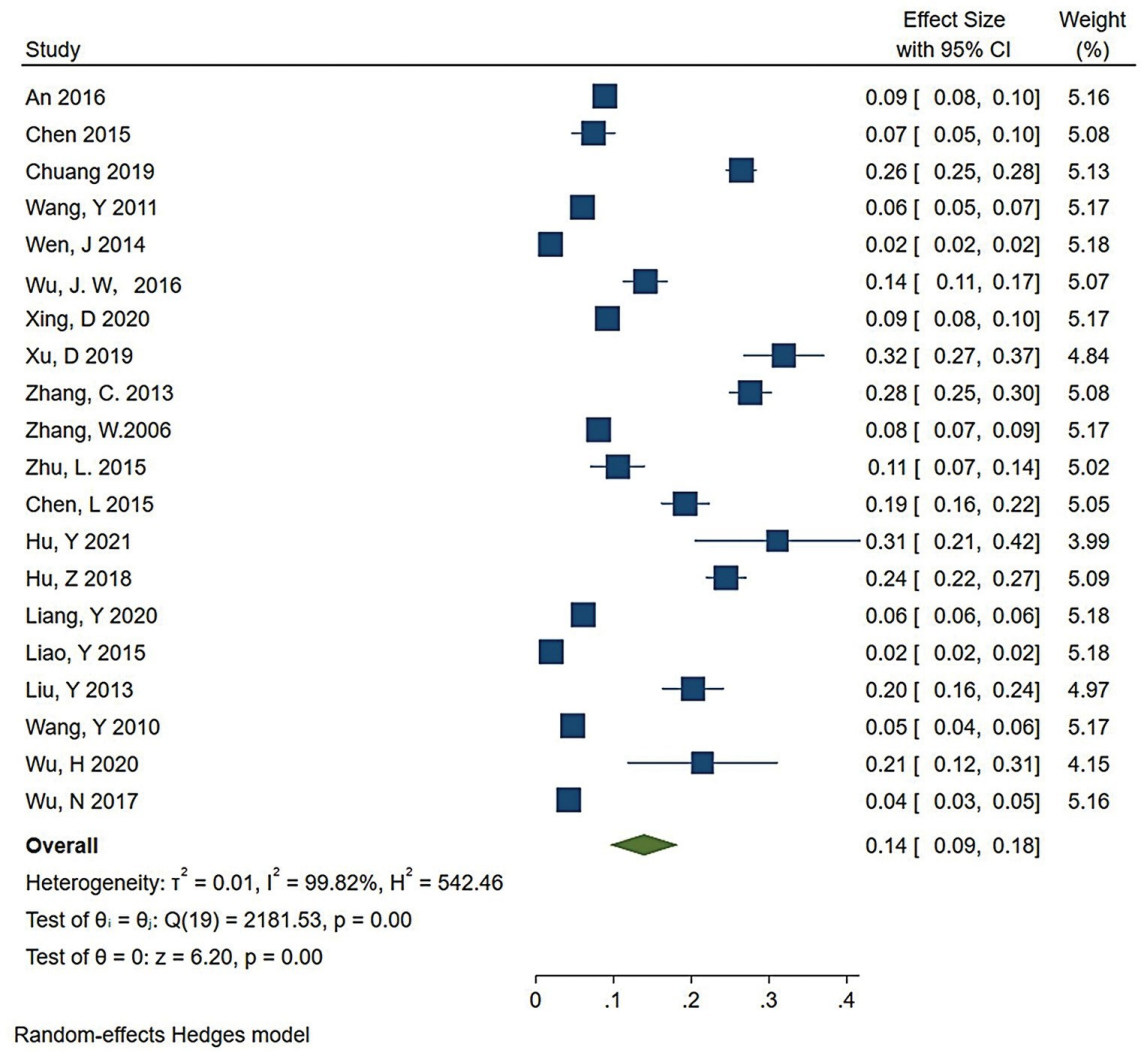
The prevalence of OAB in Chinese women.

A total of 54730 individuals were included in the study, with each study’s sample size ranging from 70 to 12701. The prevalence of OAB ranged from 1.9% to 32% (Table 2). The OAB prevalence of 14% [95% Confidence Interval (CI): 0.09–0.018] in Chinese women was demonstrated by the random-effects model (Fig 2). A random-effects model was used to estimate the pooled prevalence of OAB, owing to the considerable heterogeneity observed among the studies. The heterogeneity level was 99.82%. This type of meta-analysis is constrained by high heterogeneity, which is an unavoidable aspect of meta-analyses aimed at estimating pooled prevalence [39].

Additionally, a high degree of heterogeneity was observed in the meta-analysis. The prevalence of OAB among Chinese women was significantly associated with various factors, such as age, BMI, region, and survey year (*p* < 0.001). Age, BMI, region, and survey year were analyzed in subgroup analyses to assess the potential origin of heterogeneity and the associations between the explanatory variables at the study level and the pooled estimate. The results of subgroup analysis of different age groups demonstrated that the highest prevalence was found in the 31-40-year-old group, which was 18% (95% CI: 0.08–0.28), which was higher than the ≤ 30-year-old group (13%, 95% CI: 0.07–0.20), the 51-60-year-old group (12%, 95% CI: 0.10–0.14), and the > 60-year-old group (11%, 95% CI: 0.02–0.21) and 41-50-year-old group (7%, 95% CI: 0.06–0.08). The results of subgroup analysis of different BMI segments demonstrated that the highest prevalence of 18% (95% CI: 0.05–0.31) was for the 18.5–23.9 group, which was higher than the < 18.5 group (13%, 95% CI: 0.08–0.13), the ≥ 28 group (11%, 95% CI: 0.07–0.16), and the 24–27.9 groups (10%, 95% CI: 0.05–0.31). The results of subgroup analysis in different regions displayed that the prevalence of OAB in North China and Southwest China was the highest (21%, 95% CI: 0.13–0.30; 21%, 95% CI: 0.12–0.31, respectively). The prevalence of OAB in Central China was 12% (95% CI: 0.05–0.20), while in East China, it was 6% (95% CI: 0.02–0.14). The results of subgroup analysis of different survey years demonstrated that the prevalence of OAB in < 2006 was 8% (95% CI: 0.07–0.09), 2006–2010 was 10% (95% CI: 0.02–0.22), 2011–2015 was 14% (95% CI: 0.08–0.19), and 2016–2021 was 18% (95% CI: 0.08–0.26). The subgroup analysis demonstrated that heterogeneity was not eliminated (Table 3, S1–S4 Figs).

**Table 3:** The subgroup analysis of the prevalence of OAB in Chinese women.

Subgroup	Studies	*P*	*I*^2^ (%)	Effect Model	Prevalence (95% CI)
Survey Years					
<2006	1	-	-	Random	0.08 (0.07, 0.09)
2006–2010	4	<0.001	99.92	Random	0.10 (0.02, 0.22)
2011–2015	8	<0.001	98.91	Random	0.14 (0.08, 0.19)
2016–2021	7	<0.001	99.67	Random	0.18 (0.08, 0.26)
Age					
≤30	6	<0.001	98.46	Random	0.13 (0.07, 0.20)
31–40	5	<0.001	97.93	Random	0.18 (0.08, 0.28)
41–50	3	0.84	0	Random	0.07 (0.06, 0.08)
51–60	3	0.98	0	Random	0.12 (0.10, 0.14)
>60	3	<0.001	95.8	Random	0.11 (0.02, 0.21)
BMI					
<18.5	4	0.02	0	Random	0.10 (0.08, 0.13)
18.5–23.9	4	<0.001	97.64	Random	0.13 (0.04, 0.22)
24–27.9	4	<0.001	94.55	Random	0.18 (0.05, 0.31)
≥28	3	0.21	0	Random	0.11 (0.07, 0.16)
Regions					
North China	7	<0.001	97.15	Random	0.21 (0.13, 0.30)
East China	5	<0.001	99.88	Random	0.06 (0.02, 0.14)
Central China	3	<0.001	99.26	Random	0.12 (0.05, 0.20)
Southwest China	1	-	-	Random	0.21 (0.12, 0.31)

**Fig 3:**
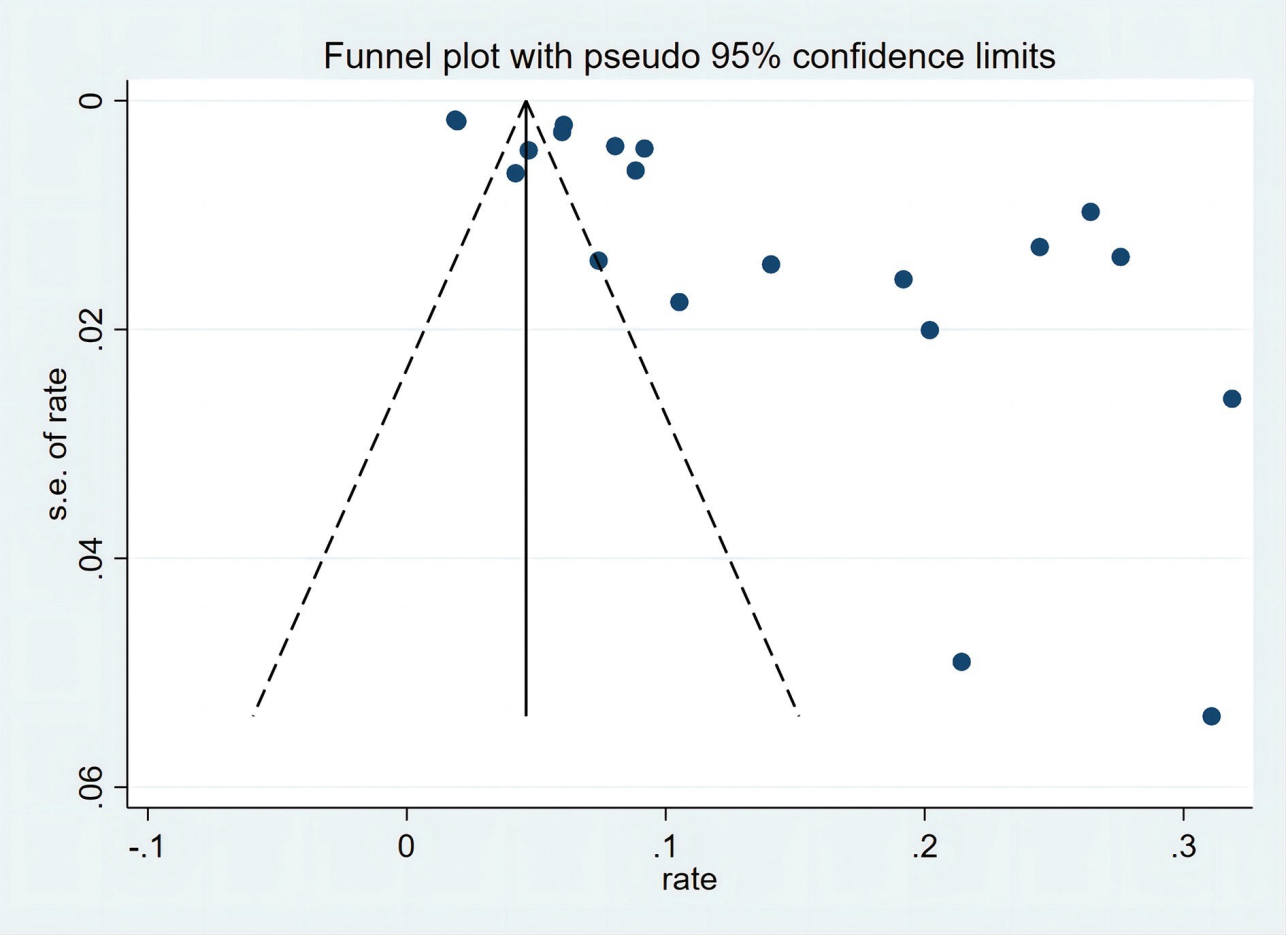
The funnel plot suggests publication bias.

A sensitivity analysis was conducted by sequentially excluding each study from the meta-analytic model owing to high heterogeneity. This finding depicted no substantial changes in the prevalence of OAB among Chinese women (S5 Fig). Publication bias was assessed by visually examining the funnel plot and using Egger’s test. The marked asymmetry in the funnel plot suggested publication bias (Fig 3), which was supported by the results of Egger’s test (p<0.001) (S6 Fig).

## Discussion

This systematic review and meta-analysis aimed to estimate the prevalence of OAB in Chinese women, delineate the epidemiological characteristics of OAB in Chinese women and identify the various characteristics of OAB in Chinese women. First, the prevalence of overactive bladder in Chinese women was 14% (95% CI: 9%–18%). It was also observed that the prevalence of OAB has significantly increased over the past decade, from 8% before 2006 to 18% at present. Second, a higher prevalence was observed among women aged 31–40 compared to the other age groups. Third, the prevalence of OAB was higher in the group with a BMI ranging from 24–27.9 than in other groups. Lastly, it was observed that North China and Southwest China exhibited greater OAB prevalence than Central China and East China.

A higher prevalence of OAB (18%, 95% CI: 8%–28%) was observed in the subgroup of women aged 31–40 compared to other age groups, as revealed by the subgroup analysis. However, the findings of the present study contradict the aforementioned research. Age is a well-known risk factor [40–42]. Studies conducted by Donaldson MM and Lightner DJ that the prevalence of OAB increases with age; however, aging does not seem to be considered one of the primary risk factors for OAB in Chinese women in our study [43, 44]. Several studies have demonstrated a higher incidence of symptoms in women due to reduced estrogen levels resulting from the postmenopausal state [16, 40, 45]. The results indicated that the highest prevalence of OAB was observed in the age group of 31–40 years old, although the reasons for the observed differences remain unclear. One possible reason could be the pressure exerted by marriage, family, and work [25, 46]. Some scholars have proposed the bladder-gut-brain axis theory, which suggests that urinary system and gastrointestinal tract-related symptoms may be caused by mental factors such as mental stress [47] and negative emotions [48, 49]. Additionally, multiple studies have pointed out that the occurrence of OAB is related to tract infections [50–52]. Khan Zainab’s research confirmed that patients with OAB symptoms have significant bacterial growth on enhanced urine culture, which is often undetectable through routine culture, suggesting the possibility of subclinical infection [53]. A higher likelihood of tract infection was observed in sexually active women. This may explain why Chinese women ages 31–40 are more susceptible to overactive bladders.

Based on the regions studied, we divided the studies into those conducted in North China, East China, Central China, and Southwest China. The prevalence was higher in North and Southwest China than in East and Central China, with only one study being conducted in Southwest China. However, the prevalence in men in South China was higher than that in women in South China (26.19% vs. 17.95%), whereas the prevalence in North China was the opposite (12.22% vs. 24.61%). Differences in the prevalence of OAB in China may be attributed to dietary habits. The climate in North China is dry and cold, whereas that in Southwest China is humid and cold. The consumption of alcohol and spices, such as pepper, is preferred by residents in these regions [40, 54–56], which may also be the reason for the different results. However, the specific mechanisms underlying this variation remain unclear.

Our study obtained results that differed from those of previous studies. The incidence rate was even lower in the BMI ≥ 28 group than in the BMI 18.5–23.9 and 24–27.9 groups. Previous studies have found a significant, positive correlation between BMI and the prevalence of OAB [57–62]; however, all of these studies are epidemiological in nature and therefore provide only subjective evidence for this relationship. Tariq F. Al-Shaiji confirmed that there was no significant correlation between OAB and any BMI category for most urodynamic study parameters.

In addition, China faces challenges both now and in the future. China is the most populous country in the world; therefore, even if the incidence of OAB remains constant, the number of OAB patients will continue to rise. In the United States, the cost of medical care for patients with OAB was greater than 2.5 times that of patients without OAB [63]. Furthermore, patients with OAB and chronic age-related comorbidities have higher medical costs than non-OAB controls with the same comorbidities. However, samples from the general population may underestimate the prevalence of OAB, which necessitates the development of more efficient screening methods.

This study had some limitations. First, there was still evidence of heterogeneity across studies, even though subgroup analyses were performed, which suggested that age, BMI, region, and survey year were not the main sources of heterogeneity. Second, heterogeneity was relatively high across all analyses; however, the degree of interpretability was minimal. Regarding publication bias, it is mainly due to relevant published articles that we identified were observational studies prone to bias. However, the main strength of this study is that most of the included studies had large sample sizes. Two researchers independently extracted data from the articles and reviewed them for accuracy.

## Conclusions

Over the past 20 years, the incidence of OAB has increased in Chinese women over the past 20 years, affecting more than 20% of women aged 31–40 years and older. The prevalence of OAB was not positively correlated with age and BMI. Given the increasing incidence of OAB, preventive and control measures have become even more critical.

## Supporting information

S1 Checklist PRISMA 2009 checklist used in this meta-analysis. (DOCX)

S1 Fig Forest plot for the age subgroup analysis of the prevalence of OAB in Chinese women. (TIF)

S2 Fig Forest plot for the BMI subgroup analysis of the prevalence of OAB in Chinese women. (TIF)

S3 Fig Forest plot for the region subgroup analysis of the prevalence of OAB in Chinese women. (TIF)

S4 Fig Forest plot for the survey year subgroup analysis of the prevalence of OAB in Chinese women. (TIF)

S5 Fig Sensitivity analyses for the meta-analysis of the prevalence of OAB in Chinese women. (TIF)

S6 Fig Egger’s test suggests publication bias. (TIF)

## Abbreviations

AHRQ: Agency for Healthcare Research and Quality
BMI: Body Mass Index
CBM: China Biology Medicine
CI: Confidence Interval
CNKI: China National Knowledge Infrastructure
OAB: overactive bladder
QOL: quality of life
