# Supplementary figures and images for: Prevalence of overactive bladder in Chinese women: A systematic review and meta-analysis
Source: PLoS One. 2023 Dec 21;18(12):e0290396. doi: 10.1371/journal.pone.0290396 (PMC10735185; doi:10.1371/journal.pone.0290396)

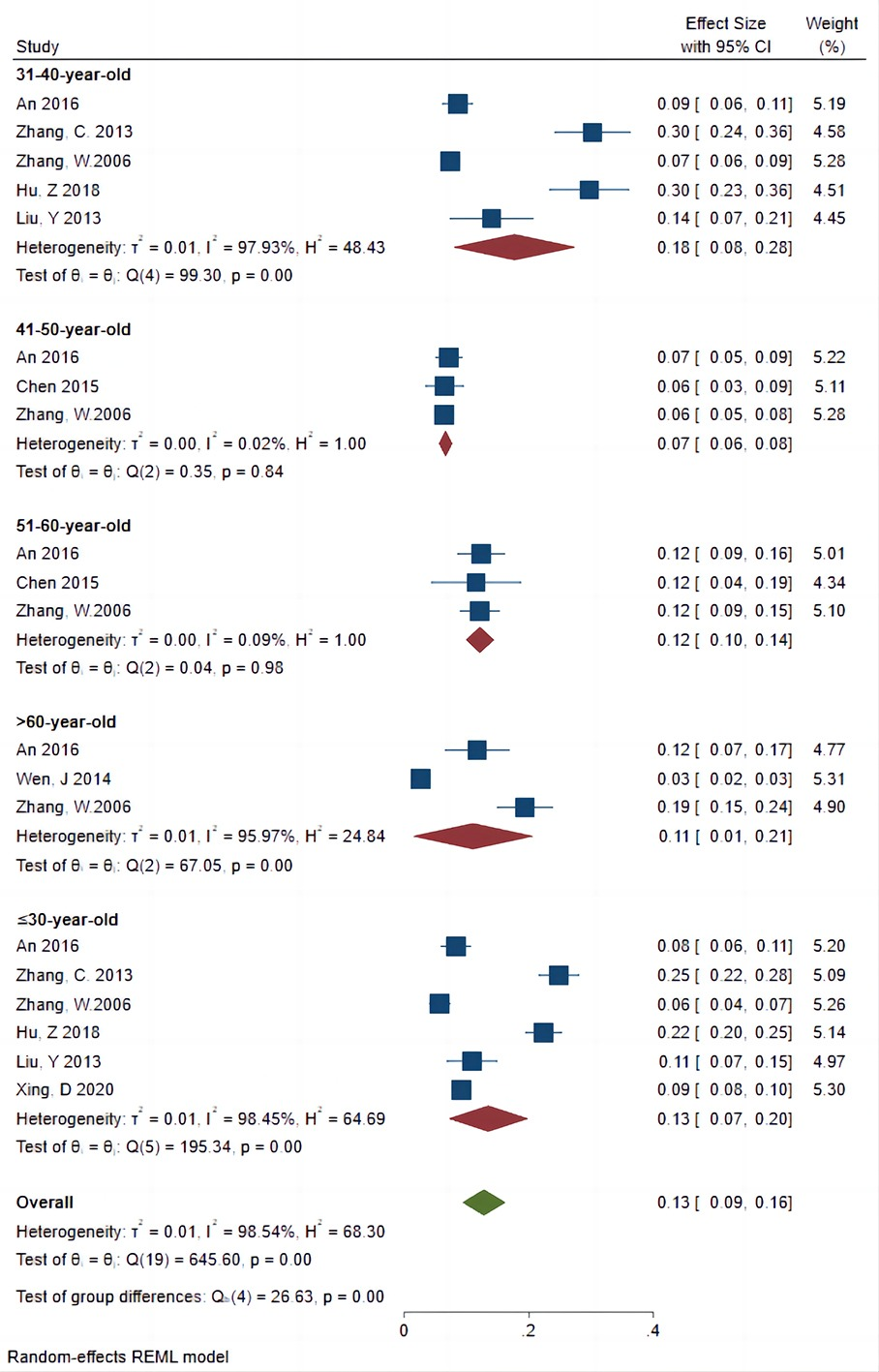

Supplement: S1 Fig — (TIF) [file pone.0290396.s002.tif]

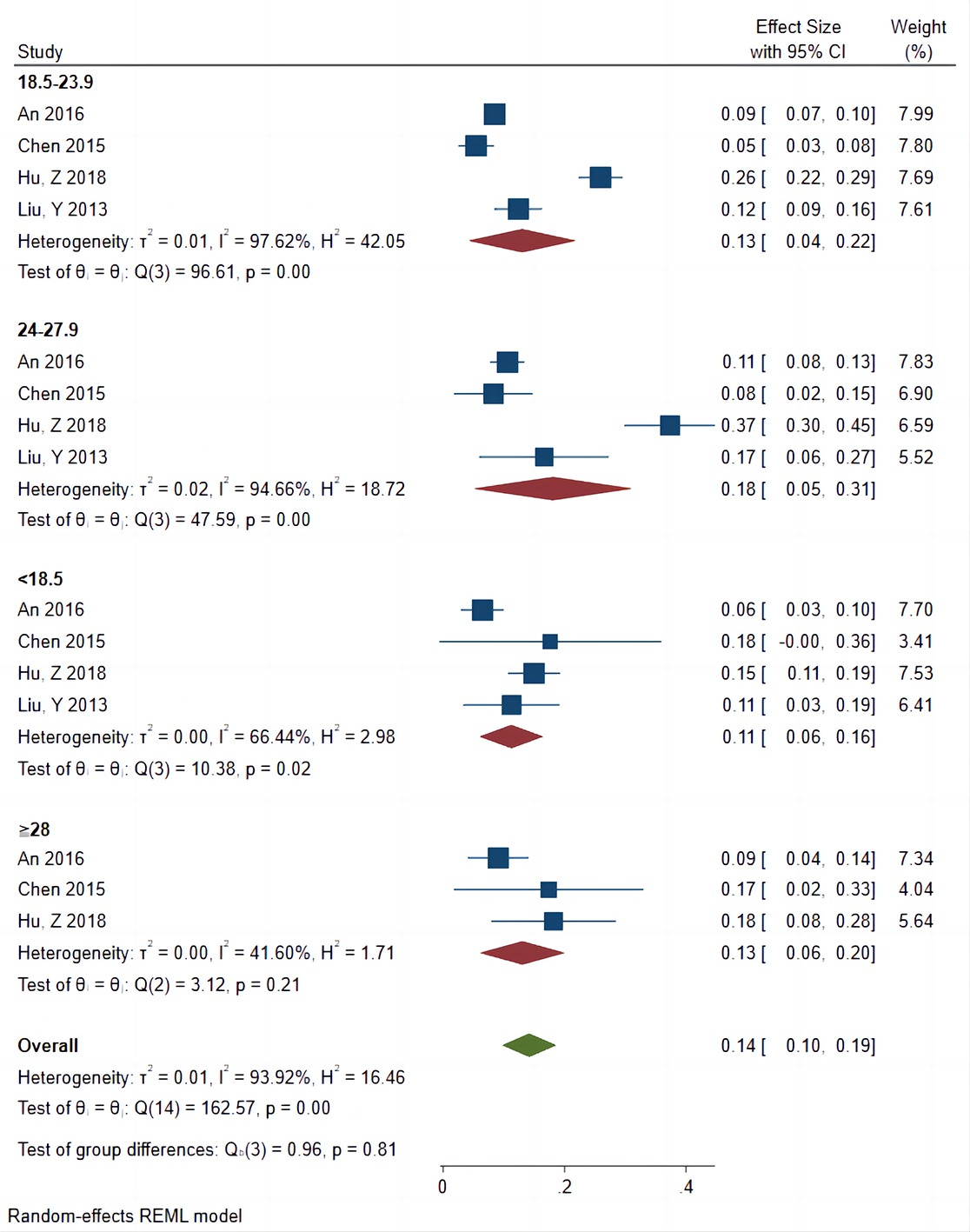

Supplement: S2 Fig — (TIF) [file pone.0290396.s003.tif]

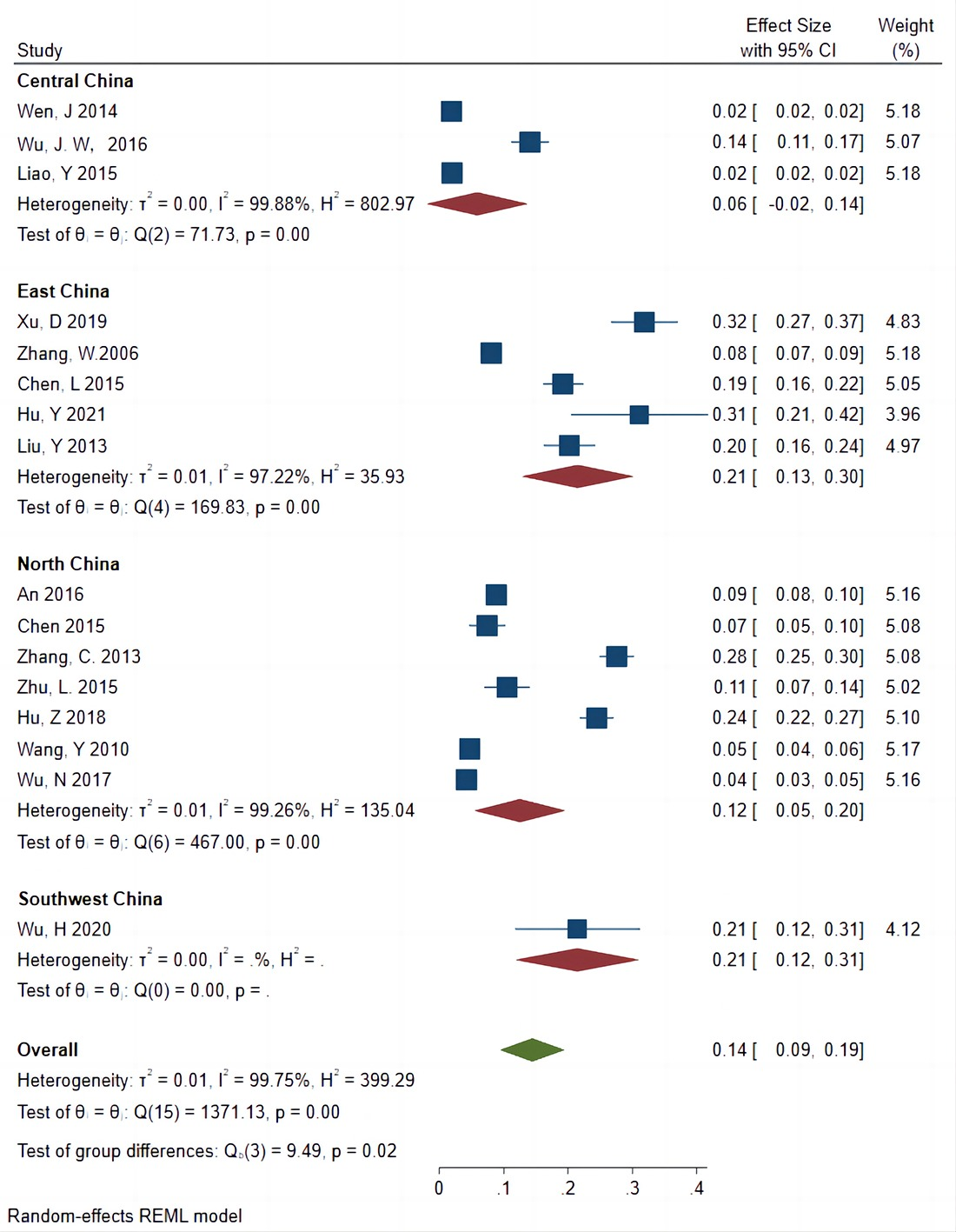

Supplement: S3 Fig — (TIF) [file pone.0290396.s004.tif]

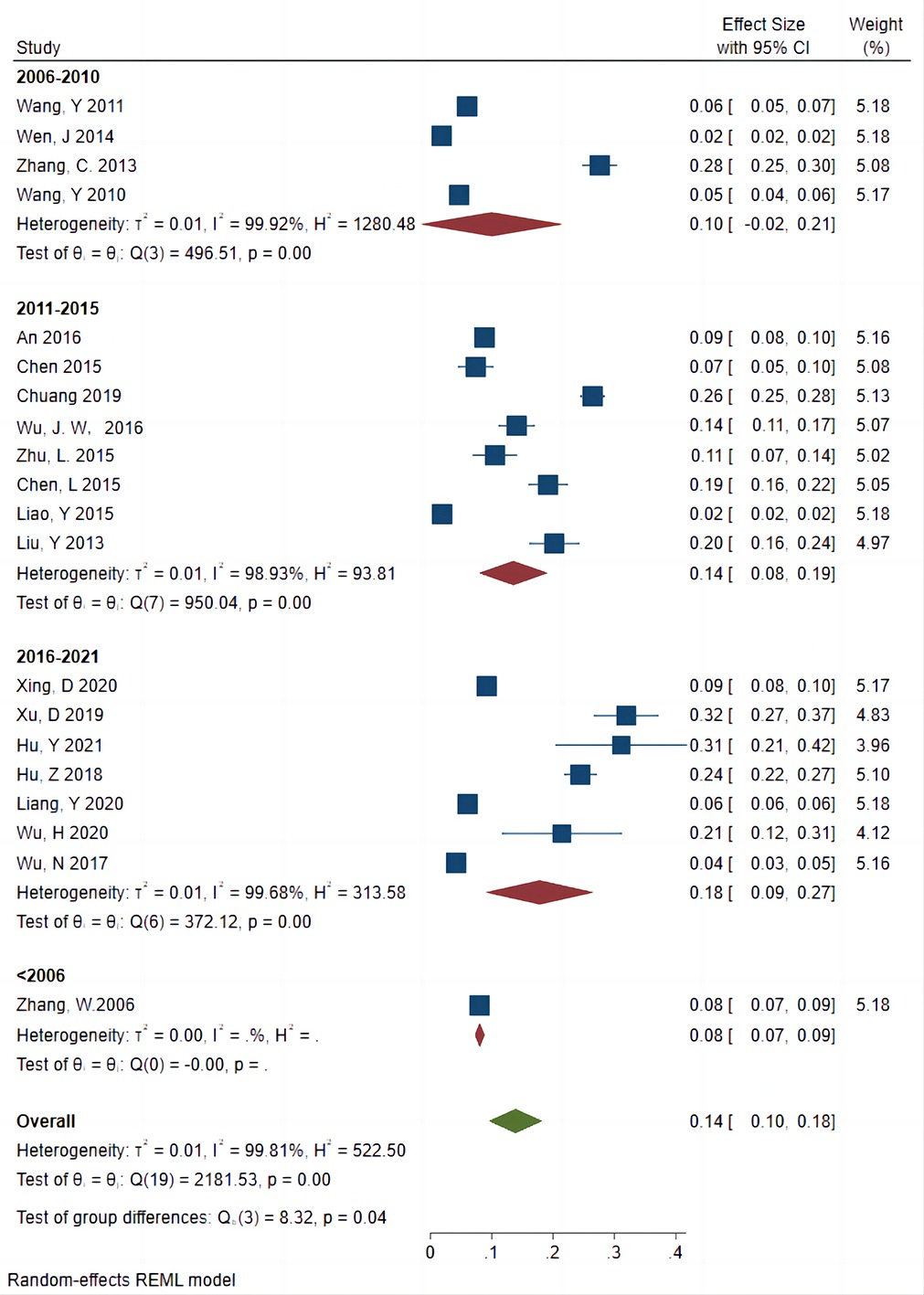

Supplement: S4 Fig — (TIF) [file pone.0290396.s005.tif]

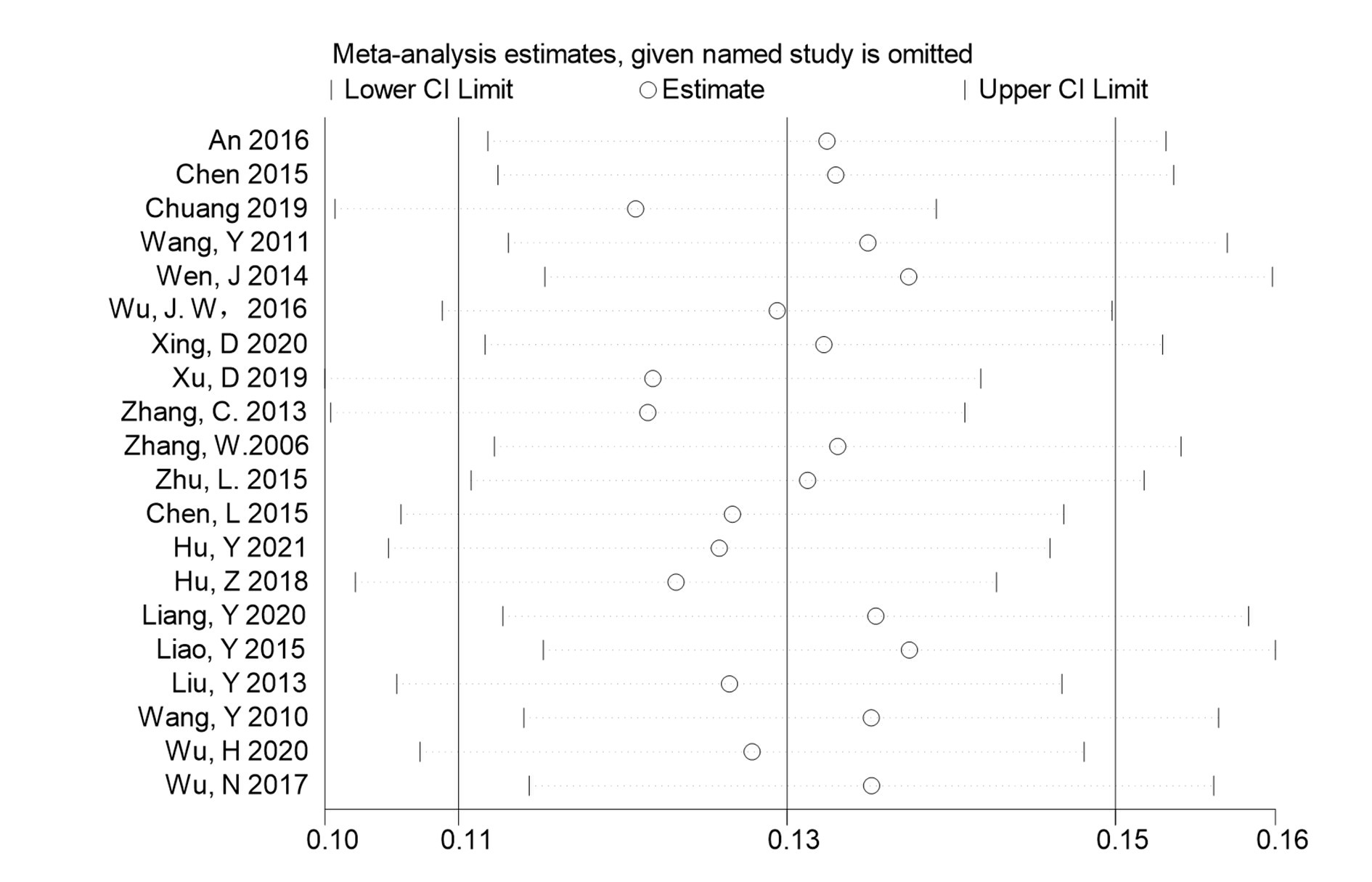

Supplement: S5 Fig — (TIF) [file pone.0290396.s006.tif]

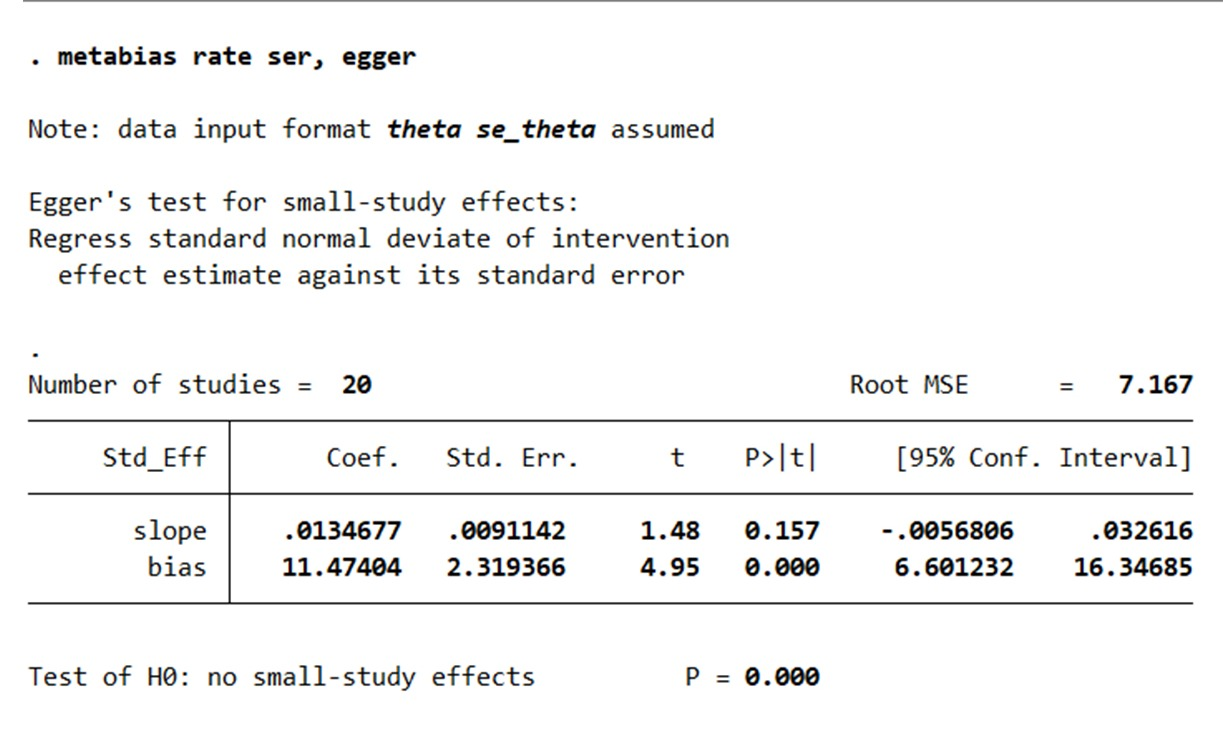

Supplement: S6 Fig — (TIF) [file pone.0290396.s007.tif]
